# Shifted windowing vision transformer-based skin cancer classification via transfer learning

**DOI:** 10.1016/j.clinsp.2025.100724

**Published:** 2025-09-06

**Authors:** Jian Lian, Lina Han, Xiaomei Wang, Zhenpen Ji, Lei Cheng

**Affiliations:** a School of Intelligence Engineering, Shandong Management University, Jinan, Shandong, China; b Department of Electrical and Automation, Shandong Labor Vocational and Technical College, Jinan, Shandong, China; c Shandong Qinlu Energy Technology Co., Ltd, Jinan, 250357, China

**Keywords:** Skin cancer, Classification, Vision transformer, Machine vision, Transfer Learning

## Abstract

**Objective:**

Skin cancer is widely recognized as one of the most perilous diseases on a global scale. Early identification of skin lesions can significantly enhance the treatment effects by aiding in clinical decision-making, hence mitigating the risk of disease progression and metastasis. Unfortunately, the skin images used for training are usually limited and imbalanced.

**Methods:**

To tackle the aforementioned challenges, a considerable number of deep learning models have been introduced for distinguishing skin cancer. And promising findings have been achieved by these algorithms. Nevertheless, the cross-domain adaptability and resilience of these models remain unresolved for actual clinical applications. One limitation of convolutional neural network-based deep models is their difficulty in finding the embeddings of global relationships for an image. This issue arises from the restricted receptive field of the convolutional operator, which primarily focuses on local information. To address this disparity, the present work introduces an innovative approach based on a vision transformer, with the aim of achieving skin cancer categorization. The presented model leverages the attention mechanism. Furthermore, transfer learning is employed to enhance the weighting parameters of the proposed model.

**Results:**

Experimental results show the superior performance of the proposed approach compared to traditional approaches, indicating the efficacy of the proposed vision transformer architecture.

**Conclusions:**

The promising results demonstrate that the proposed approach is a potentially valuable instrument for skin cancer identification.

## Highlights

• A classification method based on a vision transformer with a shifted window is proposed.
• The proposed transformer model consists of two primary blocks and an attention unit.
• The performance of this work outperforms state-of-the-art.

## Introduction

Skin cancer is a highly widespread ailment worldwide, wherein melanoma stands as the leading cause of mortality among different types of cancers. Skin cancer is also a significant public health issue, with a higher incidence rate among Americans compared to all other types of carcinomas combined. On a daily basis, a staggering number of over 9500 individuals in the United States are diagnosed with skin cancer. According to a study conducted by Cancer Research Institute in 2023,^1^ the mortality rate for this particular disease is alarmingly high, with more than two individuals succumbing to it per hour. According to recent studies,^1^^,^^2^ newly diagnosed cases of malignant melanoma over the past decade. Although early detection indeed has the potential to decrease mortality rates. The timely detection of skin cancer is crucial, as it significantly improves the likelihood of successful treatment.^3^^,^^4^ The situation is exacerbated by a global shortage of radiologists and other healthcare professionals who possess the necessary qualifications to interpret diagnostic data, particularly in underprivileged regions and emerging economies.^5^ Furthermore, the presence of a dispute within a collective of radiologists serves to further substantiate this occurrence.^6^ Moreover, the significance of efficiency and effectiveness in life-saving endeavors necessitates the utilization of machine learning-based technologies that offer prompt screening and precise diagnosis.^7^^,^^8^

The current methodologies employed for the identification of skin cancer have demonstrated a high level of proficiency, with encouraging outcomes as a result. Numerous investigations have demonstrated that machine learning-based assessments have the potential to equal or surpass the diagnostic abilities of physicians when it comes to particular skin lesion images. For instance, Esteva et al.^9^ employed a dataset consisting of 129,450 samples, including 3374 dermoscopic samples, to train a Convolutional Neural Network (CNN) model for skin cancer classification. The authors conducted a comparative analysis of their model’s performance against that of 21 dermatologists in two binary classification scenarios: distinguishing between malignant melanomas and benign nevi, and differentiating keratinocyte carcinomas from benign seborrheic keratosis. And the evaluation was based on the assessment of biopsy-proven clinical images. The initial occurrence exemplifies the most commonly occurring malignancies, whereas the subsequent incidence exemplifies the most lethal form of cancer. The study conducted by Bechelli^8^ involved an assessment of machine learning and deep learning models in the context of classifying skin cancer images. The primary finding of this study indicates that deep learning models exhibited superior performance compared to machine learning models in terms of accuracy. In a recent study conducted by^10^ an approach was introduced for the classification of melanoma and benign skin tumors. The proposed strategy involved the utilization of a stacking technique, employing three folds of classifiers. The system was trained using a dataset consisting of 1000 images of skin cancer, categorized as either melanoma or benign. Three distinct deep learning models were employed for extracting features from the images of skin cancer. In the work of,^11^ the identification of skin cancer was performed using CNN and Support Vector Machines (SVM). Accordingly, the CNN achieved an accuracy rate of 95.03 percent, while the SVM achieved an accuracy rate of 93.04 percent.

Meanwhile, the CNN-based models suffer from the inability to capture the global associations between long-range pixels in an image. To be specific, it can be attributed to the local receptive field. Receptive field in the deep neural network is used to represent the receptive range of neurons at different positions within the network to the original image.^12^ The reason why neurons are unable to perceive all the information in an image is that both convolutional^13^ and pooling^14^ layers are commonly used in the networks, with local connections between layers through sliding filters. The larger the value of the neuron’s receptive field, the larger the range of the original image it can cover, which also means that it contains more global and semantic features; The smaller the value, the more localized and detailed the features it contains. Therefore, the value of the receptive field can be roughly used to judge the level of abstraction at each level of a CNN network. On the other hand, another branch has emerged in the field of deep learning, which is the self-attention mechanism-based transformer models. Transformer is a popular model in Natural Language Processing (NLP). Following the early work of Attention is All You Need,^15^ it has swept over a variety of NLP sectors with its apparent results. Recent works in the field of computer vision have begun to leverage transformers, e.g. the Vision Transformer (ViT).^16^ ViT divides the input image into patches (size of 16 × 16), and projects each patch as a vector of fixed length. The subsequent encoder operation is identical to the original Transformer.^15^ Due to the classification of images, a class token is appended to the input sequence, and the corresponding output of the class token is taken as the final category outcome. Accordingly, ViT has been applied in skin cancer classification due to its characteristics. For instance, a two-stage pipeline for the categorization of skin cancer was proposed by Aladhadh et al.in their article.^17^ To boost the data samples in the first step, a collection of data augmentation operations was utilized. And by separating the input images into consecutive patches, the ViT model designed for medical images was utilized. Finally, the classification result was generated using the Multi-Layer Perceptron (MLP) module. The application of ViT for skin cancer categorization was then assessed by Nikitin and Shapoval in the study.^18^ Arshed et al.^19^ suggested a method for categorizing various forms of skin cancers based on ViT. This study also addressed the problem of class imbalance in the dataset for skin cancer.

Generally, the deep learning methods rely on a substantial quantity of image samples and employ convolution operations to train the models and extract local embeddings, respectively. On the other hand, the skin cancer datasets that are publicly available often exhibit imbalanced class distributions, with unequal occurrence counts for each class. Consequently, traditional augmentation techniques are commonly utilized when there is an insufficient quantity of images available for training a deep CNN. To shed light on sample insufficiency, an open dataset was exploited in this study. Sequentially, a set of data augmentation techniques was incorporated to enlarge the quantity of the skin cancer images in this dataset. By adding slightly modified copies of training samples, rather than capturing new images, data augmentation techniques can artificially boost the number of images.^20^ In addition, the strategy of transfer learning was then employed to eliminate the impact of skin cancer image imbalance and insufficiency on the performance of image classification.

Bearing the above-mentioned analysis in mind, this work introduces a novel vision transformer approach for the purpose of skin cancer classification. The self-attention mechanism is employed in the suggested transformer model to control the global associations among long-range pixels. The computational complexity of self-attention has a quadratic relationship with image resolution. Consequently, the direct implementation of the original vision transformer model^16^ would be impractical, particularly when dealing with high-resolution images. In addition, the provision of additional memory capacity and a substantial allocation of processing resources would be inevitable. To address this disparity, the vision transformer employed in this study incorporates a sliding window operation, resulting in a notable reduction in parameters and computational resources. Furthermore, the utilization of pre-trained weighting values is employed for the purpose of skin lesion classification utilizing the ImageNet dataset.^21^ The performance evaluation of the proposed approach utilizes the publicly available dataset International Skin Imaging Collaboration (ISIC),^22^, ^23^, ^24^ comprising eight distinct types of skin lesions. Furthermore, a comparison analysis was conducted to examine the proposed strategy in relation to state-of-the-art procedures. Empirical evidence demonstrates that the proposed methodology exhibits superior performance compared to the current leading approach, as evidenced by a range of evaluation metrics.

In general, this study’s contributions are as follows:

• For the categorization of skin cancer, a pipeline based on a vision transformer with a shifted sliding window has been presented.
• Two primary blocks, along with the attention unit, make up the suggested transformer model, which is used to sequentially deal with the regular and shifted regions around an image.
• Results from experiments show that the presented technique performs better than the state-of-the-art algorithms.

The subsequent sections of this article are organized as follows. The proposed transformer model is comprehensively elucidated in The suggested transformer model is fully described in Section 2. The experimental methodology and the corresponding results obtained from the experiments, along with the supporting analysis and discussion, are presented in Section 3. Ultimately, the investigation culminates in the presentation of its conclusion, which may be found in Section 4.

## Materials and methods

**Fig. 1:**
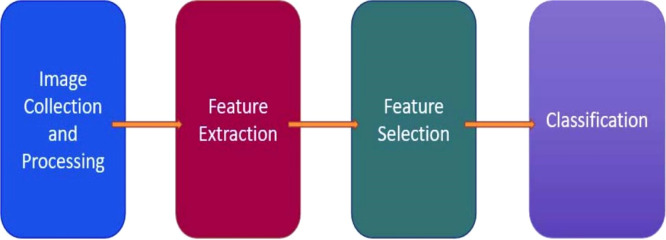
The workflow of a typical machine learning system.

In a conventional classification procedure within the context of machine learning, several standard stages are involved, including pre-processing, feature extraction, feature selection, and classification. These stages have been widely utilized in numerous applications based on artificial intelligence, as depicted in Fig. 1.

The limited availability of biological pattern data necessitates the reliance on manual feature sets in numerous classification techniques. However, these feature sets have inherent limitations in their ability to effectively generalize to dermoscopic skin images. The lesions exhibit a high degree of similarity in terms of color, size, shape, and texture, resulting in strong interconnections and limited availability of distinctive features. Hence, the endeavor to categorize skin lesions by manual feature-based methodologies is deemed to be ineffectual. In contrast, deep learning algorithms are designed to autonomously extract the most optimal characteristics from data samples. When comparing shallow networks to deep networks, particularly CNN models, it becomes evident that the latter are more effective in revealing intrinsic features for precise image classification. However, the extraction of appropriate embeddings heavily depends on the quantity of training samples, which was rarely utilized to its full potential in most of the previous studies. The approach described in this study was the integration of pre-trained models that had been trained on a large dataset of skin cancer.

### Dataset and data augmentation

Due to the uneven quantity of images in every single category, the ISIC 2019 dataset^22^, ^23^, ^24^ has been identified as one of the most challenging to classify skin cancer images into eight categories. In this dataset, the total number of images is 25,331, with a resolution of 256 × 256 and RGB color space. It comprises Actinic Keratosis (AK) 867, Basal Cell Carcinoma (BCC) 3323, Benign Keratosis (BKL) 2624, Dermatofibroma (DF) 239, Melanoma (MEL) 4522, melanocytic Nevus (NV) 12,875, Squamous Cell Carcinoma (SCC) 628, and Vascular Lesion (VASC) 253. In this study, the dataset was divided into three distinct components: training, validation, and testing, with training constituting 70 % of the dataset, validation comprising 20 %, and testing constituting 10 %.

Deep learning models rely heavily on extensive datasets and exhibit enhanced generalization capabilities as the volume of data increases. In order to provide data augmentation in this study, many operations are utilized, including rotation, flipping, random cropping, brightness and contrast adjustment, pixel jittering, aspect ratio manipulation, random shearing, zooming, and vertical and horizontal shifting. Data augmentation is a technique that can be employed to artificially augment the available data volume. This is achieved by integrating slightly altered versions of previously acquired training data, rather than procuring entirely new data. The purpose of this practice is to enhance the diversity of the dataset through the introduction of modest modifications to existing data instances or the generation of fake datasets based on pre-existing data. The size of the training dataset is deliberately augmented, or measures are taken to safeguard the model from overfitting from the outset, through techniques like as data augmentation or oversampling.

### Details of the backbone

**Fig. 2:**
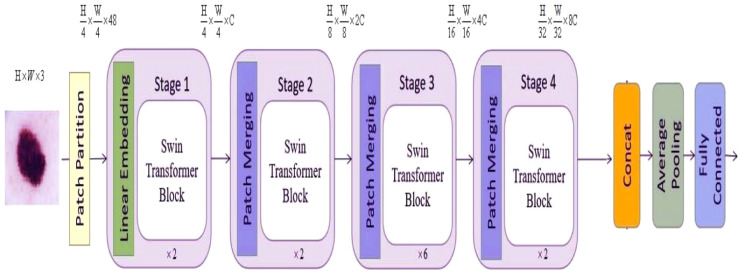
The fundamental architecture of the vision transformer model that was developed.

The flexibility of the system allows for adjustments in both the quantity of blocks and the dimensions of the tokens, enabling the system to cater to different applications, as illustrated in Fig. 2. During the input operation, the images are divided into non-overlapping patches, each measuring 4 by 4. The feature dimension of an individual patch is 48, calculated as the product of the dimensions 4 × 4 × 3, where 3 denotes the number of RGB channels. The characteristic of each patch is determined by the concatenation of the pixel values within the patch, which is analogous to the approach employed by the vision transformer.^16^

In the first stage, the original feature is projected upon a fixed dimension by means of a linear embedding layer. Subsequently, a sequence of swin transformer blocks^25^ is employed, comprising two separate self-attention units. In addition to this, it is ensured that the number of tokens in each block remains unchanged, specifically at a value equivalent to the linear embedding unit $\frac{H}{4}\times \frac{W}{H}$: . The method proposed draws on patch merging components to effectively reduce the feature sizes by a factor of two, hence enabling the construction of a structured presentation. Stage 2 encompasses the initial patch merging module and feature transformation, a process that is then reiterated during Stages 3 and 4. Furthermore, there is a rise in the resolution of the output elements from Stage 1 to Stage 4, as outlined below, $\frac{H}{4}\times \frac{W}{4}\times C,\;\frac{H}{8}\times \frac{W}{8}\times C,\;\frac{H}{8}\times \frac{W}{8}\times C,\;\frac{H}{16}\times \frac{W}{16}\times C$, and $\frac{H}{32}\times \frac{W}{32}\times C$ represent a series of calculations using the variables H and C. The hierarchical representation is a significant differentiating factor between the Swin Vision Transformer proposed in^25^ and the original Vision Transformer introduced by.^16^ This differentiation is established by the combined implementation of Stage 2, Stage 3, and Stage 4. In conclusion, the output vector is constructed using global average pooling combined with a fully connected layer. The dimensions of the output vector are determined by the formula $N=\frac{H}{32}\times \frac{W}{32}$. The linear classifier only considers the topmost C elements of the output vector.

### Swin transformer block

**Fig. 3:**
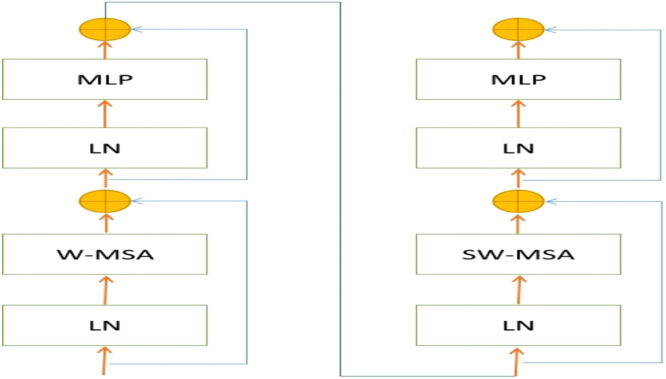
The constituent modules that constitute the Swin Transformer model. Layer normalization, commonly abbreviated as LN, refers to a technique used in the field of machine learning and neural networks. The abbreviations W-MSA and SW-MSA are used to represent the multi-head self-attention modules with conventional and shifted apertures, respectively.

As depicted in Fig. 3, it is evident that each stage comprises several Swin Transformer blocks, whereby each block consists of two successive Swin Transformer modules. Referring to the Multi-head Self-Attention (MSA) technique, the W-MSA and SW-MSA modules are implemented using a normal window mechanism and a shifted-window mechanism, respectively.

The following numerical equations can be employed to articulate the mathematical representation of the sequential swing transformer modules.

(1) $${\hat{z}}^{1}=W-MSA\left(LN\left(z^{l-1}\right)\right)+z^{l-1}$$

(2) $$z^{l}=MLP\left(LN\left({\hat{z}}^{1}\right)\right)+\hat{z}$$

(3) $${\hat{z}}^{l+1}=SW-MSA\left(LN\left(z^{l}\right)\right)+z^{l}$$

(4) $$z^{l+1}=MLP\left(LN\left({\hat{z}}^{l+1}\right)\right)+{\hat{z}}^{l+1}$$

where the term W-MSA denotes window-based multi-head self-attention, MLP is an abbreviation for multi-layer perception,^26^ SW-MSA represents shifted-window multi-head self-attention, and LN stands for layer normalization as described in the work of.^27^

### Shifted window mechanism

The MSA module, based on a window mechanism, as opposed to the preliminary vision transformer that employed global self-attention and necessitated the computation of associations between each token and all other tokens, uses a window of size *M* × *M*, where the default value of M is 7. This approach aids in limiting the computational workload by reducing the volume of information that requires processing. Hence, the introduction of the window-based self-attention mechanism makes the computational complexity more feasible compared to the quadratic complexity of the vision transformer^16^ which is dependent on the image resolution h×w.

(5) $$\Omega \left(MSA\right)=4hwC^{2}+2{\left(hw\right)}^{2}C$$

(6) $$\Omega \left(W-MSA\right)=4hwC^{2}+2M^{2}hwC$$

where M is equal to 7 in the settings that follow.

**Fig. 4:**
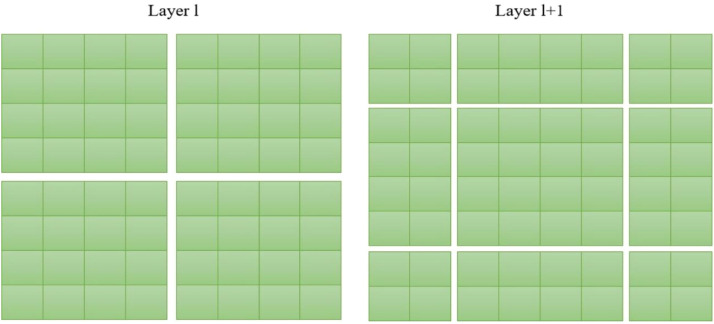
The illustration of the SW-MSA mechanism employed in the proposed methodology.

Furthermore, the SW-MSA technique is specifically devised to enhance the encoding of the overall interconnections among the pixels contained within every window. The use of SW-MSA enables the optimization of the interplay between multiple windows to its fullest extent. The implementation of the partitioning technique for the normal window may be observed in layer l, as depicted in Fig. 4. Within each window, self-attention is computed. The window partitioning is displaced both horizontally and vertically in the subsequent layer, leading to the emergence of a more diverse range of windows. Consequently, the computation of self-attention in Layer *l* + 1 involves the traversal of the windows that existed in Layer l.

It should be noted that the loss function employed in the suggested model is a cross-entropy loss. This loss is computed by comparing the ground truth category of the image with the classification output generated by the proposed technique, as depicted in Fig. 2.

### Transfer learning

Transfer learning refers to the methodology employed in machine learning, whereby an algorithm is initially trained on a certain dataset and afterwards utilized on a distinct dataset, referred to as an assignment that is closely associated with the original task. The terms “domain adaptation” and “transfer learning” are used to describe these occurrences. These notions are employed to facilitate the process of generalization within a different environment. Transfer learning is a highly effective approach in deep neural networks, even in the presence of substantial data and resource requirements. The available data sets are not optimal for training deep neural networks from inception due to their limited diversity of images resulting from a minimum number of included images. Transfer learning may be seen as a potential approach to address this problem.^28^

This work leverages transfer learning to differentiate between eight distinct categories of skin cancer images. The pre-trained models employed in this research are sourced from ImageNet, a dataset initially employed for classifying a diverse range of 1000 items.^21^ To facilitate the process of transfer learning, it is important to adhere to three distinct protocols. To commence, a sequential adjustment is made to the final trainable layer of each neural network individually, with the purpose of discerning images depicting skin cancer. Furthermore, the weights and biases of the first layers are adjusted in order to preserve the generalization extraction capacity of such layers. In summary, enhancing the learning process for the underlying layers might be achieved by increasing the learning rate coefficients for both the weights and biases.

To note that the authors conducted this diagnostic study following STARD guidelines.^29^

## Experimental results

### Implementation details

The experiments were conducted employing four NVIDIA RTX 3080 GPUs and the PyTorch framework.^30^ The chosen backbone structure in the developed paradigm is the Swin-T vision transformer. The input images undergo a process of resizing, resulting in a consistent resolution of 224 pixels for both width and height. Furthermore, the authors incorporated the pre-trained weighting values obtained from ImageNet^21^ as part of the initialization process for the proposed vision transformer. In addition, a batch size of 16 was selected, the Adam Optimizer was employed as the hyper-parameter, a learning rate of 1e-4 was chosen, a depth of 8 was specified, and the number of epochs was set to 500. Finally, a 10-fold cross-validation procedure is employed in the comparative experiments to guarantee the accuracy and dependability of the findings.

The following equations contain the assessment criteria of accuracy, precision, recall, and F1 score to evaluate the performance of the proposed model and the comparison methodologies.

(7) $$\text{Accuracy}=\frac{TP+TN}{TP+TN+FP+FN}$$

(8) $$\text{Precision}=\frac{\text{TP}}{TP+FP}$$

(9) $$\text{Recall}=\frac{TP}{TP+FN}$$

(10) $$F1=\frac{2\times \mathrm{Pr}ecision\times \text{Re}call}{\mathrm{Pr}ecision+\text{Re}call}$$

Where the variables TP, TN, FP, and FN denote the respective counts of true positive, true negative, false positive, and false negative.

To be specific, Accuracy can be misleading in imbalanced skin cancer datasets, where the model might over-predict the majority class. Precision is crucial as it gauges the reliability of positive predictions; low precision could lead to unnecessary invasive procedures and patient distress. Recall directly impacts patient outcomes by ensuring the detection of cancerous lesions, minimizing the risk of missed malignancies. The F1 score offers a balanced assessment, integrating precision and recall to capture the model’s overall effectiveness. Meanwhile, the AUC of the ROC curve provides a comprehensive measure of the model’s ability to distinguish between cancerous and non-cancerous skin lesions across varying classification thresholds, highlighting its discriminatory power.

### Ablation study

**Fig. 5:**
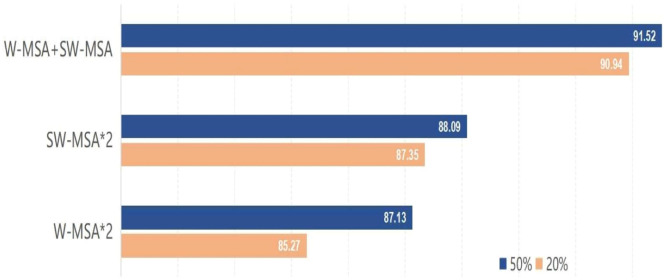
Accuracy comparison of the proposed model with respect to two different ratios (20 % and 50 %) of the training set.

In order to validate the efficacy of the newly proposed vision transformer, a series of ablation experiments were undertaken using a publicly available dataset. These experiments involved testing the introduced models under different configurations, replacing the original settings that were used. The subsequent swin transformer block employed unique configurations of blocks, as depicted in Fig. 5. In this study, the Swin Transformer block utilized three distinct combinations of the W-MSA and SW-MSA modules. These combinations consisted of two instances of the W-MSA module, two instances of the SW-MSA module, and a mixture of both the W-MSA and SW-MSA modules.

As illustrated in Fig. 5, it is evident that the suggested strategy achieves enhanced outcomes through the use of optimal configurations. The transformer model, when applied to a subset comprising 20 % of the leveraged dataset, has demonstrated superior performance compared to the other two options, exhibiting improvements of 6.65 % and 4.11 % respectively. Furthermore, the transformer model that was proposed exhibited superior performance compared to the vision transformer version on 50 percent of the identical dataset, with improvements of 5.04 percent and 3.89 percent, respectively. Consequently, the provided model was selected as the fundamental basis for further inquiries.

Furthermore, the proposed model was assessed using varying configurations of both the parameter C and the number of layers. This evaluation was conducted on a subset comprising 50 % of the training set. The specific parameters used for this evaluation are presented in Table 1. The ideal configuration within this context can be identified as *C* = 192 and the number of layers as 2, 2, 18, 2.

**Table 1:** Different settings of the backbones used for the proposed approach (on 50 % of the training set).

**Model**	**C**	**N° of layers**	**Accuracy ( %)**
Model 1	96	2, 2, 6, 2	88.41
Model 2	96	2, 2, 12, 2	89.38
Model 3	96	2, 2, 18, 2	89.56
Model 4	128	2, 2, 6, 2	90.39
Model 5	128	2, 2, 12, 2	90.68
Model 6	128	2, 2, 18, 2	90.43
Model 7	192	2, 2, 6, 2	91.19
Model 8	192	2, 2, 12, 2	91.27
Model 9	192	2, 2, 18, 2	91.52

### Experimental results

**Fig. 6:**
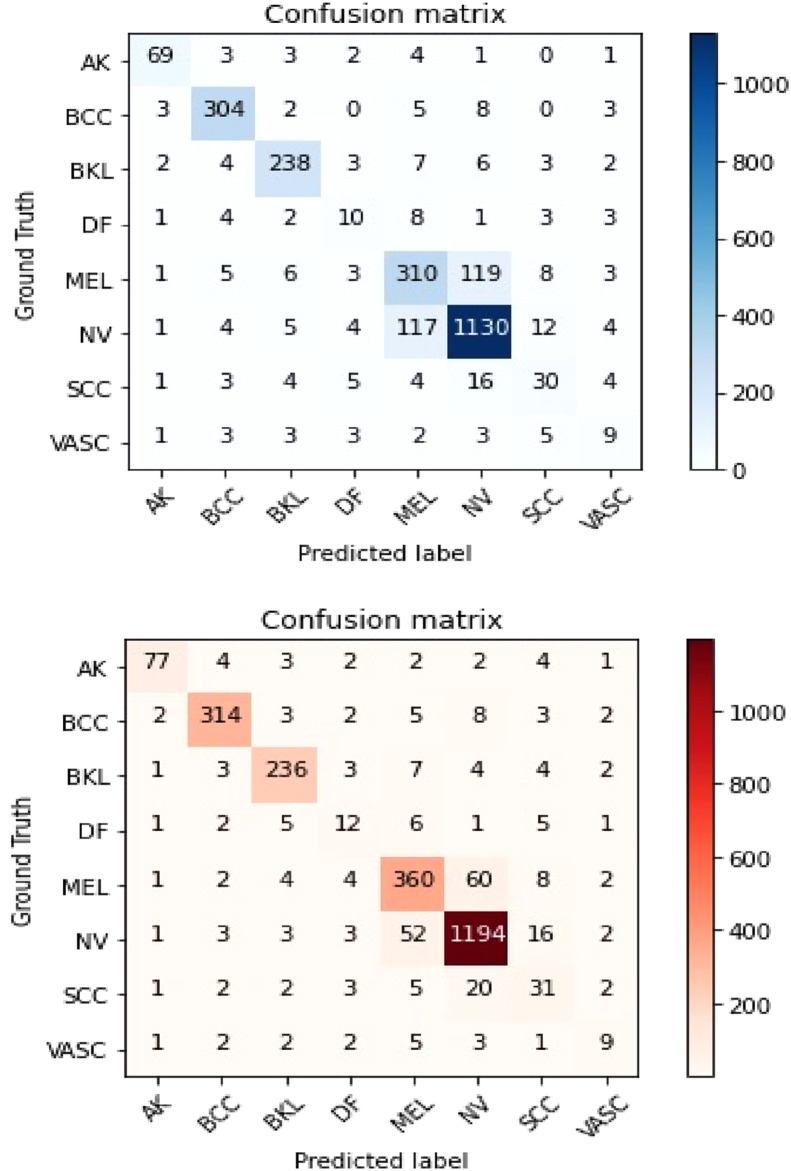
The confusion matrices pertaining to the proposed technique on the dataset that has been presented (Top) Prior to employing transfer learning, and (Bottom) subsequent to implementing transfer learning.

In addition, the confusion matrix depicted in Fig. 6 presents a comparison of the performance achieved by the suggested approach both prior to and following the use of transfer learning.

**Fig. 7:**
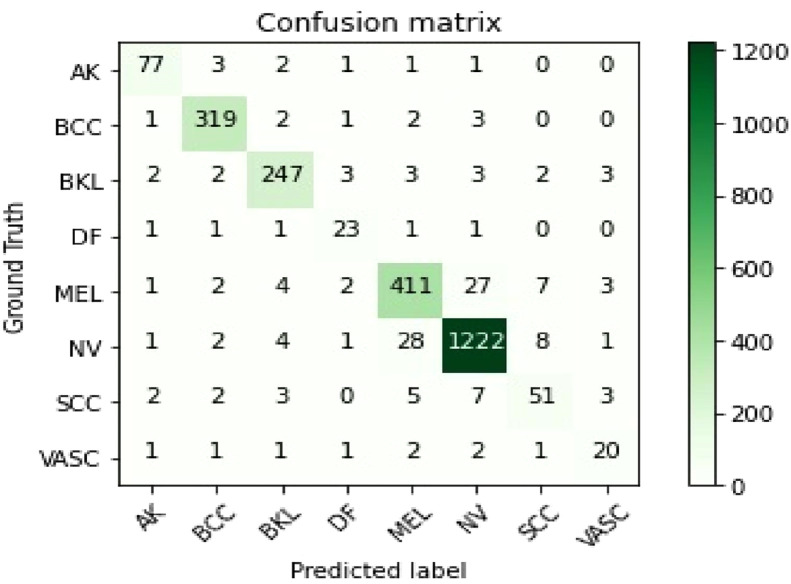
The confusion matrix of the proposed approach on the presented dataset after using data augmentation + transfer learning.

The performance comparison of the suggested approach after including data augmentation and transfer learning is presented in the confusion matrix shown in Fig. 7.

In order to fairly evaluate the performance of the proposed approach, comparison experiments were conducted with state-of-the-art methods in the field. These methods include AlexNet,^31^ GoogleNet,^32^ VGG,^33^ ResNet,^34^ EfficientNet,^35^ Inception V3,^36^ MobileNet,^37^ vision transformer,^16^ CI-Net^38^ and ours in the classification task. The comparison experiments were conducted specifically in the context of the classification task.

As seen in Table 2, the suggested classification approach exhibits superior accuracy, precision, recall, and F1 score compared to the most recent methods. To provide specific details, the method exhibits an overall accuracy improvement 15 % of 3.35 % when compared to the CI-Net. Furthermore, the proposed approach demonstrates enhanced Precision, Recall, and F1 score by 3.03 %, 6.13 %, and 4.58 % respectively, in comparison to the CI-Net. Additionally, when compared to the original vision transformer,^16^ the proposed approach showcases increased accuracy, Precision, Recall, and F1 score by 6.61 %, 4.41 %, 7.56 %, and 5.98 % respectively. In summary, the suggested approach demonstrates higher performance compared to both CNN-based and vision transformer-based algorithms, hence showcasing its prospective capability for feature extraction.

**Table 2:** Comparative analysis of the current state of the art and the proposed method.

**Method**	**Accuracy ( %)**	**Precision ( %)**	**Recall ( %)**	**F1 score ( %)**
AlexNet	69.81	67.91	71.25	69.54
GoogleNet	74.29	65.71	70.32	67.94
VGG	72.62	70.19	71.64	70.91
ResNet	75.48	72.15	72.92	72.53
EfficientNet	73.42	72.03	71.55	71.79
Inception V3	79.68	81.42	81.38	81.40
MobileNet	84.77	86.04	83.95	84.98
Vision Transformer	87.39	88.16	86.12	87.13
CI-Net	88.15	86.34	85.28	85.81
Our method	93.60	90.38	86.10	88.06

**Fig. 8:**
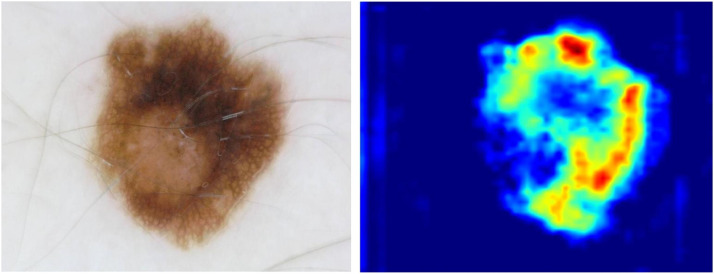
An image of skin cancer and the heatmap generated by using Grad-CAM.

Finally, to provide the interpretability of the proposed model for skin cancer classification, the technique of Gradient-weighted Class Activation Mapping (Grad- CAM) was exploited, as shown in Fig. 8.

Fig. 8 intuitively demonstrates the degree of attention that the proposed model pays to various regions when processing a skin cancer image.

## Discussion

Most CNN-based deep learning models possess the capability to extract feature maps from images. However, it is hypothesized that by including a deeper network structure, these models will exhibit enhanced proficiency in extracting features from images. Nevertheless, the performance of CNNs may be limited due to the fact that the CNN’s convolutional module primarily focuses on the local receptive fields of images. This is a contributing factor to the high success rate of CNNs. This leads to the disregard of the dependencies that exist between pixels in an image that are significantly far from each other. Meanwhile, enhancing deeper CNN models necessitates a larger allocation of processing resources.

**Fig. 9:**
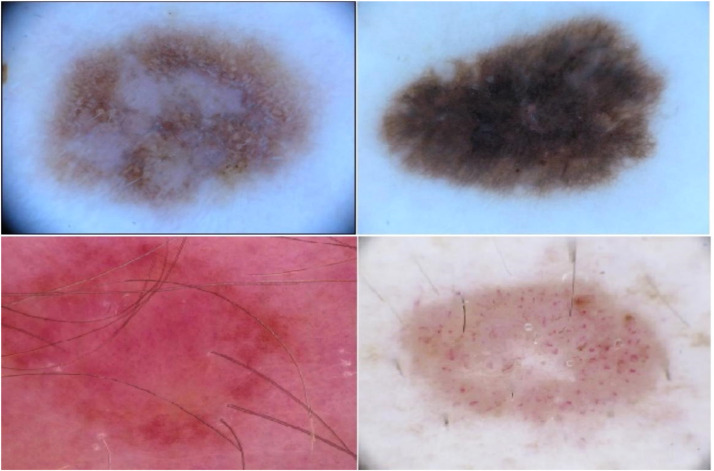
A set of sample images containing the lesions across the entire image.

In the visual representations of skin cancer, the areas that exhibit the presence of lesions are frequently distributed over the entire image, rather than being localized to a specific section of the image, as exemplified in Fig. 9. The use of additional layers in CNN models does not guarantee enhanced image classification performance, as the local receptive field mechanism may not effectively handle this image type. Consequently, this study proposes a vision transformer-based model for image classification, aiming to use the inherent links among distant pixels found inside the images. The proposed methodology consistently captures the correlation among image patches by employing the MSA mechanism. Therefore, the method being presented demonstrates the capability to retain pertinent information that aids in the process of classification. The Swin Vision Transformer, in comparison to the conventional notion of the vision transformer, demonstrates the capability to extract significant information from images while concurrently reducing computational resource demands.

However, there are some limitations associated with this study. To be specific, one restriction that affected the effectiveness of the suggested strategy was the inconsistency of the image samples in the dataset used during the trials. Moreover, the self-attention mechanism in the proposed approach, which enables the model to capture global relationships in images, demands substantial computational resources, due to the quadratic complexity of calculating attention scores with respect to the sequence length. In the context of skin cancer classification, where real-world clinical applications may involve resource-constrained devices such as mobile devices or portable imaging equipment, these computational challenges pose significant barriers to deployment.

## Conclusion

The objective of this study is to classify skin cancer images by employing a network architecture that is founded on vision transformers. In comparison to the existing state-of-the-art methods, the performance of the vision transformer model was seen to be superior. The efficacy of the proposed model is substantiated by empirical evidence derived from the dataset.

In real-world clinical applications, skin cancer classification models often encounter cross-domain challenges, such as variations in image acquisition devices, lighting conditions, patient demographics, and imaging protocols across different clinical settings. The proposed model addresses these issues through multiple mechanisms. The attention mechanism in the vision transformer plays a crucial role in cross-domain adaptability. Unlike traditional convolutional neural networks that mainly focus on local features, the self-attention mechanism allows the model to capture global relationships within skin images. This enables the model to better handle variations in image appearance caused by differences in acquisition conditions. Furthermore, the authors employ transfer learning to enhance the model’s resilience in cross-domain scenarios. By pre-training the model on a large-scale and diverse dataset that encompasses various skin image characteristics from different sources, the model can learn generalizable features. Then, fine-tuning the model on the target clinical dataset further adapts these features to the specific characteristics of the new domain. This two-step approach helps the model bridge the gap between the source and target domains, improving its performance when applied to different clinical environments.

Recently, there has been notable progress in machine vision tasks through the utilization of vision transformers. Consequently, in the foreseeable future, there will be an increased focus on conducting further research pertaining to multimodal and multi-label deep learning models in order to enhance the accuracy of skin cancer classification and prediction. In addition, the applications of deep learning models in clinical scenarios will be another direction of future work.

## Data availability

The dataset International Skin Imaging Collaboration (ISIC) 2019 used in this study can be downloaded from https://api.isic-archive.com/ collections/?pinned=true.

## Ethics statement

This study used deep learning methods to classify skin images, with the aim of identifying skin cancer types in the images. The images used in the study were from a publicly available dataset, ISIC 2019. This study did not report Human participants/human data with health outcomes, and did not involve human participants or human data. This research did not involve experimentation on animals. In addition, this study did include images showing humans or parts of humans. However, the medical images used in this study were from a publicly available dataset. Therefore, ethics approval and consent were not collected in the ethics and consent section of my manuscript.

## CRediT authorship contribution statement

**Jian Lian:** Conceptualization, Methodology, Software, Data curation, Writing – original draft, Writing – review & editing. **Lina Han:** Visualization, Investigation, Software, Validation, Writing – original draft, Writing – review & editing. **Xiaomei Wang:** Software, Validation, Writing – original draft, Writing – review & editing. **Zhenpen Ji:** Formal analysis, Validation, Visualization, Writing – review & editing. **Lei Cheng:** Formal analysis, Investigation, Validation, Visualization, Writing – review & editing.

## Declaration of competing interest

The authors declare that the research was conducted in the absence of any commercial or financial relationships that could be construed as a potential conflict of interest.
